# Spectrum Analysis Enabled Periodic Feature Reconstruction Based Automatic Defect Detection System for Electroluminescence Images of Photovoltaic Modules

**DOI:** 10.3390/mi13020332

**Published:** 2022-02-19

**Authors:** Jiachuan Yu, Yuan Yang, Hui Zhang, Han Sun, Zhisheng Zhang, Zhijie Xia, Jianxiong Zhu, Min Dai, Haiying Wen

**Affiliations:** School of Mechanical Engineering, Southeast University, Nanjing 210096, China; 230208026@seu.edu.cn (J.Y.); yangyuancsi@163.com (Y.Y.); zhanghuihui@seu.edu.cn (H.Z.); 230218029@seu.edu.cn (H.S.); ddt@seu.edu.cn (M.D.); wenhy@seu.edu.cn (H.W.)

**Keywords:** defect detection, computer vision, spectrum analysis, photovoltaic module, electroluminescence image

## Abstract

Electroluminescence (EL) imaging is a widely adopted method in quality assurance of the photovoltaic (PV) manufacturing industry. With the growing demand for high-quality PV products, automatic inspection methods based on machine vision have become an emerging area concern to replace manual inspectors. Therefore, this paper presents an automatic defect-inspection method for multi-cell monocrystalline PV modules with EL images. A processing routine is designed to extract the defect features of the PV module, eliminating the influence of the intrinsic structural features. Spectrum domain analysis is applied to effectively reconstruct an improved PV layout from a defective one by spectrum filtering in a certain direction. The reconstructed image is used to segment the PV module into cells and slices. Based on the segmentation, defect detection is carried out on individual cells or slices to detect cracks, breaks, and speckles. Robust performance has been achieved from experiments on many samples with varying illumination conditions and defect shapes/sizes, which shows the proposed method can efficiently distinguish intrinsic structural features from the defect features, enabling precise and speedy defect detections on multi-cell PV modules.

## 1. Introduction

### 1.1. Background

The world has shown considerable progress in solar energy harvesting utilizing photovoltaic (PV) technology [1,2]. The conversion efficiency and durability of PV modules are major concerns in the PV manufacturing process, which can be massively impacted by local defects on modules, such as cracks and breaks [3,4]. Therefore, an efficient defect detection method for PV products is very important for the practical use of solar energy.

A PV module is composed of many PV cells arranged in a grid layout with busbars connecting them. There are three main types of PV material: monocrystalline, polycrystalline, and thin-film technology [5,6,7]. In our case, the PV module is manufactured using a monocrystalline cell, which is made of a single pure silicon ingot. Some large defects, such as breaks, can be directly visible from the surface, while many small cracks are intrinsic, and hence cannot be captured by ordinary CCD cameras. Various non-destructive inspection techniques have been proposed to locate both extrinsic and intrinsic defects of PV modules [7], e.g., thermography (LIT) [8,9] and photoluminescence (PL) [10,11]. Among these methods, electroluminescence (EL) [12,13,14,15] imaging is one of the most adopted techniques, which has the advantages of a higher resolution and more accurate localization of microcracks and printing problems.

The EL image of a PV module involves the conversion of electrical energy into emitted near-infrared light. Current is fed into a PV module in the dark and light emission is captured by infrared-sensitive camera. Areas with no defects have a higher conversion efficiency and appear brighter in the resulting image. Deficiencies like cracks, breaks, and speckles reduce current passage, leading to dark spots or lines in the image. With an EL imaging system, PV modules are characterized as grayscale images, which can be inspected visually. To increase efficiency and reliability, and reduce cost, the industry needs automated defect detection with computer vision to replace labor-intensive manual examination.

### 1.2. Related Work

A great deal of research has been dedicated to automated defect detection, localization, and/or classification of PV modules [15]. Schuss et al. [16] used thermographic techniques to characterize defect regions. Wang et al. [17] used an image processing pipeline and adaptive thresholding using both adaptive color threshold and window size. This method works on a single PV cell that is rectangular shaped of varying sizes. Deitsch et al. [18] managed to segment a large PV module into multiple PV cells. Curve estimation with subpixel precision is utilized on local ridge information to extract dividing grids. This method could break a large-scale detection problem into smaller ones within a standardized PV cell. Akram et al. [19] proposed a light CNN architecture trained on the labeled dataset from Deitsch’s work. This is a supervised learning method, which is highly dependent on an adequate amount of labeled data. Moreover, the trained model cannot trivially transfer to another product that has different shapes and structures. Dhimish el al. [20] used Fourier transformation and band filtering to remove noise, but the method could not distinguish intrinsic layout features from defect features. Our work is most similar to the research of Tsai et al. [21]. They used Fourier transformation to remove the influence of inhomogeneous texture in the background. This method has an assumption of the defect shape, and hence is able to detect some specified defects. Tsai’s work aimed to remove the defects to produce a reference image, while in this paper, the aim is to remove all features other than the defects. In addition, Tsai’s method was designed on a polycrystalline cell, which contains an inhomogeneous background texture, while in our case, the monocrystalline cell is nearly homogeneous within one silicon slice.

### 1.3. Problem Description

**Figure 1:**
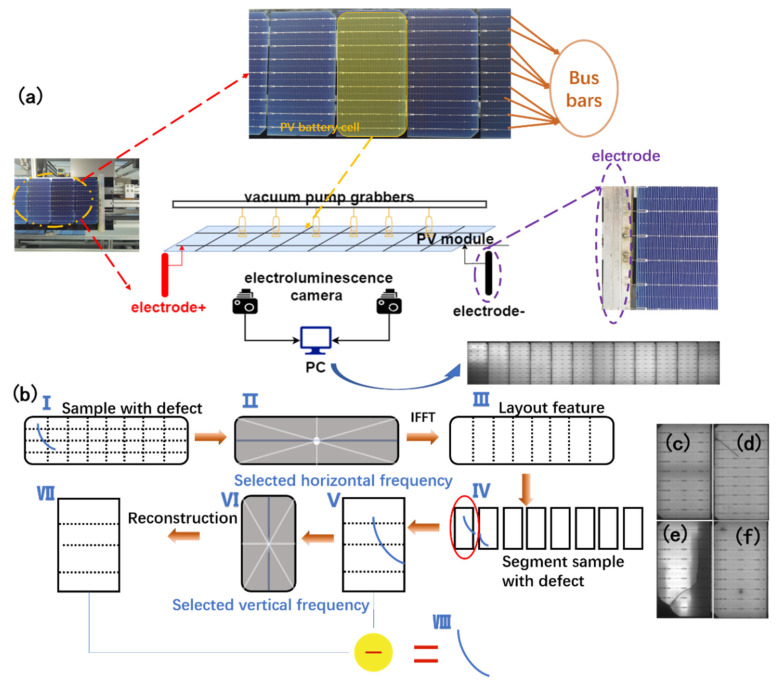
(**a**) The EL imaging system of a PV module inspection pipeline, which activates the EL effect on each module and converts infrared light into grayscale images; (**b**) the spectrum domain analysis-based method to exploit the periodic features and locate the defects; (**c**) normal PV cell in a defect-free module; (**d**) PV cell with cracks; (**e**) PV cell with cracks and breaks; (**f**) PV cell with speckles.

The research target is a PV module composed of multiple PV cells (usually 10–12 cells) aligned in a row. The PV cell in this study was a printed silicon wafer soldered along 9 busbars. The PV module outputs 200 W of power in optimal conditions, with 21.4 V of open circuit voltage. Figure 1a shows the original product captured by an ordinary CCD camera. The PV module under study is supplied with 24 V DC power, which is slightly higher than the rated voltage, to activate the electroluminescence effect, where infrared light is emitted from the PV module. The light was then captured using an EL sensor, producing a grayscale image, as shown in Figure 1a. Defects, such as cracks, breaks, and speckles, are characterized as dark areas with different shapes, as shown in Figure 1c–f. Cracks appear as thin lines or groups of lines, but do not divide PV cells completely, such as in Figure 1d. Breaks appear as shape of polygons, which are usually insulated cell parts. Speckles are circle or spot-like areas, which usually result from polluted silicon. The aim was to design a system to locate all specified defect types in a whole EL of a PV module. There are several challenges that need be resolved in our method. Firstly, the illumination of the EL image is not across the whole image. As a result, some PV cells may appear darker or brighter than others in the same image, which makes it impossible to find a global threshold. Secondly, the PV module is not aligned perfectly, so the image may appear slightly translated or rotated, which makes it harder to generate a self-reference template with a fixed positional boundary. Thirdly, though the basic shapes and layouts of the PV modules are identical, individual variations may appear for many reasons.

## 2. Proposed Method

Fourier transformation is a very effective tool to analyze periodic features. PV cells can be regarded as repetitive signals along the horizontal direction. Within a PV cell, busbars can be seen as repetitive signals along the vertical direction. From prior knowledge about the structure of PV modules, the frequency can be determined by counting the occurrence of repetitive patterns. Repetitive signals will have concentrated energy on the major frequency and its harmonics in the spectrum domain; however, the defect features are, most times, sparse in the image, which will be scattered across the spectrum. In our proposed method, a two-stage Fourier analysis is carried out. First, vertical dividing lines are reconstructed from the filtered Fourier spectrum of the threshold binary image of the whole EL image to segment the PV modules into PV cells. Second, the PV cell is reconstructed with only the busbars through vertical filtering of the Fourier spectrum. The defects can hence be extracted by eliminating reconstructed features from all features. Preprocessing is required to extract an ideal impulsive 2D signal of the dividing lines and busbars so that Fourier transform could produce a clearer spectrum.

### 2.1. Global Feature Extraction

The input image as shown in Figure 1a is 3000 × 500 pixels in size, with silicon wafers appearing as brighter gray rectangles. Spaces between cells, busbars, and defects appears as darker areas. A binarization process could extract all interesting features according to the characteristics of the defects. However, PV cells are not perfectly identical to each other, so light emission can vary. The energy distribution within one slice is not homogeneous, with centers usually brighter and two ends darker. In addition, the captured gray level can be affected by the environment, camera settings, and other influences. 

In this study, an adaptive threshold is applied, in which a threshold is selected for each window. For each pixel $P_{i,\;j}$, a K × K window is selected around the pixel, the mean value of the window is calculated as Equation (1):

(1) $${\mathrm{mean}}_{i,j}=\frac{1}{K^{2}}\underset{m,n}{\sum }p_{m,\;n}\;$$

where m,n represents a pixel within the window. Constant C is selected so that:

(2) $$B_{i,j}=\{\begin{array}{c} 1,\;\mathrm{if}\;P_{i,j}<{\mathrm{mean}}_{i,j}-C\;\\ 0,\;\mathrm{elsewise} \end{array}$$

Window size K is selected as the minimal square to contain a local feature. In this study, K = 21 is selected so that 21 × 21 windows are considered locally homogeneous in normal areas. Constant C is selected according to the requirement of detection. The larger the C value, the more features are extracted, and with higher noise. The resulting image will have all features of interest in white, and the remaining areas in black. In our case, C = 15 was selected so that most defect features can be extracted with minimal noise. It was noticeable that some edge features were obscured, especially at the two ends of the PV module. This is a result of the unstable electroluminescence effect close to the electrode. However, in the following Fourier analysis, the slight erosion of the edge features could be robustly reconstructed, with little effect on the detection performance.

### 2.2. Fourier Spectrum Filtering

The Fourier theorem states that any signal can be decomposed as the sum of sinusoid functions with different amplitudes and frequencies. An image is a two-dimensional function of pixel value according to space coordinate. Let the function of an image be f(x,y), which represents the pixel value at position (x,y) in the image. The two-dimensional discrete Fourier transform is defined as:

(3) $$G\left(m,\;n\right)=\frac{1}{\mathrm{MN}}\overset{M-1}{\underset{x=0}{\sum }}\overset{N-1}{\underset{y=0}{\sum }}f\left(x,\;y\right)e^{-j2\pi \left(\mathrm{xm}/M+\mathrm{yn}/N\right)}$$

where M, N is the width and height of the image. With the spectrum, the original signal can be recovered by simply summing the sinusoids given by G(m,n). However, the goal is to filter the spectrum so that only the desired frequencies are remained to reconstruct the image in the spatial domain. In our case, the extracted feature image contains both structural layout edges and defects. The Fourier analysis is adopted to form an ideal feature image without defects. There are two characteristics being utilized to filter the spectrum. First, the layout features are repetitive in (almost) vertical and horizontal directions; second the defects are sparse in the image. With the approximate prior knowledge of the structure which is the number of times the pattern is repeated and the direction, the proposed method can easily select the desired spectrum. The magnitude of repetitive patterns will concentrate on the frequencies equaling to the number of repeating and its harmonics. Moreover, the defect features which are not periodic will have its magnitude scattered across the spectrum. In the rest part of this chapter, the proposed method will be illustrated with theoretic analysis, simulations and actual testing in real PV module images.

#### 2.2.1. Theoretic Analysis

In the general equation of discrete Fourier transformation shown in Equation (3), the complex exponential is a periodic component. Given enough periods, the sum of this factor is close to zero unless the exponent is zero. Let the original function  f(x,y)  also be a 2D sinusoid function which can be written as complex exponentials according to Euler equation.

(4) $$f\left(x,\;y\right)=\sum \frac{A}{2}[e^{-j2\pi \left(\frac{\mathrm{px}}{M}+\frac{\mathrm{qy}}{N}\right)}+e^{j2\pi \left(\frac{\mathrm{px}}{M}+\frac{\mathrm{qy}}{N}\right)}]\;$$

where A is the amplitude of frequency p, q. The discrete Fourier transformation of a 2D sinusoid is:

(5) $$\begin{array}{cc} ℱ\{f(x,y)\}= & G\left(m,\;n\right)=\frac{1}{\mathrm{MN}}\sum_{x=0}^{M-1}\sum_{y=0}^{N-1}f(x,\;y)e^{-j2\pi \left(\frac{\mathrm{xm}}{M}+\frac{\mathrm{yn}}{N}\right)}\\ & =\frac{A}{2\mathrm{MN}}\sum_{x=0}^{M-1}\sum_{y=0}^{N-1}[e^{-j2\pi \left(\frac{\mathrm{px}}{M}+\frac{\mathrm{qy}}{N}\right)}+e^{j2\pi \left(\frac{\mathrm{px}}{M}+\frac{\mathrm{qy}}{N}\right)}]\\ & \cdot e^{-j2\pi \left(\frac{\mathrm{xm}}{M}+\frac{\mathrm{yn}}{N}\right)}\\ & =\frac{A}{2\mathrm{MN}}\sum_{x=0}^{M-1}\sum_{y=0}^{N-1}[e^{-j2\pi \left(\frac{\left(p+m\right)x}{M}+\frac{\left(q+n\right)y}{N}\right)}\\ & +e^{j2\pi \left(\frac{\left(p-m\right)x}{M}+\frac{\left(q-n\right)y}{N}\right)}] \end{array}$$

In Equation (5), the first complex exponential term always has a non-zero exponent, hence it always sums to a near zero value. The exponent that might be zero is  (p−m)x+(q−n)y. Only when m == p and n == q, does the magnitude have a non-zero value in the Fourier transformation results. This is how Fourier transform detects a sinusoid frequency. However, the image is often not a straightforward sinusoid-like signal. Most times, images are not even periodic. In this study, the layout edges are periodic, just like a 2-D rectangular wave; the defects are non-periodic. The Fourier transform of a non-periodic function is the transformation of the periodic extension of that function, supposing that the signal is repeated only once in the sample.

An image can be seen as the sum of many feature images. Using Fourier theory, the Fourier transform of the sum of two functions is the sum of the Fourier transform of each. This can be proved through the definition of Fourier transform (Equation (6)):

(6) $$\begin{array}{cc} ℱ\left\{g\left(t\right)+h\left(t\right)\right\} & =\int_{-\infty }^{\infty }g\left(t\right)e^{-i2\pi \mathrm{ft}}\mathrm{dt}+\int_{-\infty }^{\infty }h\left(t\right)e^{-i2\pi \mathrm{ft}}\mathrm{dt}\\ & =G\left(f\right)+H\left(f\right)\; \end{array}$$

In the proposed method, the image is regarded as the sum of periodic features and non-periodic features. A periodic function that is not a sinusoid, such as the layout edges in the PV module image, can be regarded as the sum of many sinusoid functions. The component with the same frequency as the function has the highest magnitude, while other frequencies have a smaller magnitude. If only the frequency with the maximum magnitude, which is just the frequency of the original function, and its harmonics are selected, the original function can be reconstructed with very little difference. This is the theoretic basis that can be used to reconstruct the main layout edge features via Fourier analysis of the PV module. The non-periodic features in the image are extended as a periodic function that repeats once in the sample. From Equation (3), a sparse nonperiodic feature that has a non-zero value in only a few points, will sum up to a much smaller value compared to a periodic feature. Moreover, the magnitude distribution will concentrate at a frequency of around 1 Hz.

One important thing to explain is why the proposed method has to use Fourier transform compared to inverse transformation since the repeating time of the desired feature must be known in advance. With known frequencies, an image can indeed be built by just summing a few sinusoids; however, the image of the PV module often has stochastic translation and rotation, so the phase information must be obtained to precisely reconstruct the image. The Fourier transform reflects the phase of the function by imaginary parts, while the frequency and magnitude remain unchanged no matter the phase. This can be proved in Equation (7):

(7) $$\begin{array}{cc} ℱ\left\{g\left(t+\theta \right)\right\}= & \int_{-\infty }^{\infty }g\left(t+\theta \right)e^{-i2\pi \mathrm{ft}}\mathrm{dt}\;=\int_{-\infty }^{\infty }g\left(u\right)e^{-i2\pi f\left(u-\theta \right)}\mathrm{du}\;\\ & =e^{-i2\pi f\theta }\int_{-\infty }^{\infty }g\left(u\right)e^{-i2\pi \mathrm{fu}}\mathrm{du}\;=e^{-i2\pi f\theta }G\left(f\right) \end{array}$$

#### 2.2.2. Simulations

**Figure 2:**
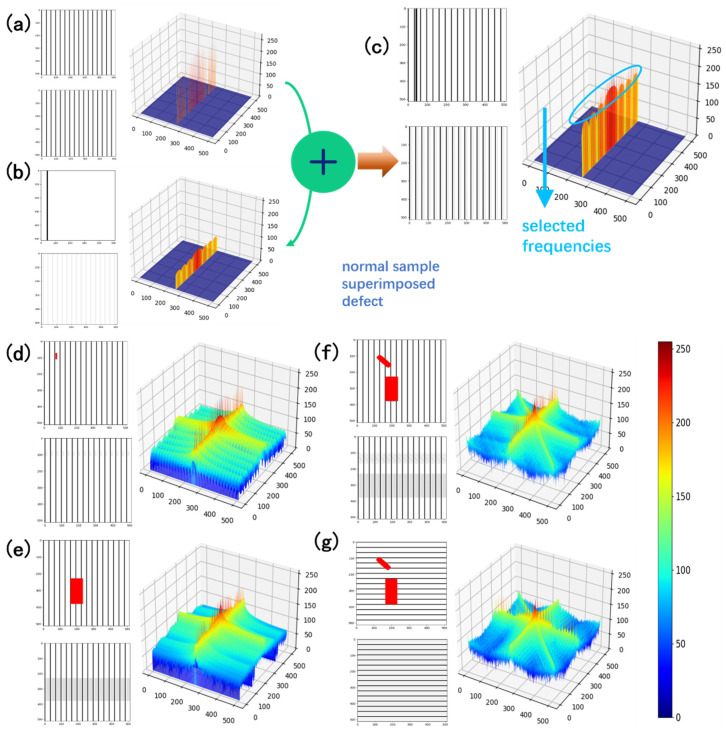
Simulation of the proposed method. Fourier transform spectrum magnitude and reconstructed image after spectrum filtering of (**a**) normal stripe feature, the spectrum is concentrated values along the horizontal axis; (**b**) only defect feature, the spectrum energy is scattered along the horizontal axis; (**c**) normal feature and defect combined, the spectrum is the sum of first two; (**d**) small speckle defect; (**e**) large area defect; (**f**) multiple defects; (**g**) defects on horizontal stripes, filtered with only vertical frequencies; defects can hardly be seen in the reconstructed image although the original defect size is big.

In this section, several synthetic images are used to demonstrate the proposed method. The base image is a 512 × 512 empty image with regular stripes. The stripes repeat in the horizontal direction 16 times. The Fourier transform of the image is a few dots that are placed with a fixed gap, horizontally, as shown in Figure 2a. The center point of the spectrum reflects the overall energy of the original image, which should be maintained in the filtering process. Other points in the spectrum reflect the frequency and magnitude of the features. A high value in the spectrum indicates that a periodic feature of that frequency is in the original image. From the spectrum, it can be observed that the first peak is 16 units always from the center point, which is the frequency of the stripes. There are a few harmonics with a gradually lower magnitude, which are the sinusoids to approximate the non-sinusoid function. Figure 2b shows a synthetic defect, which is a stripe apart from the periodic ones. The Fourier spectrum shows that the magnitudes are scattered, with decentralized energy. Then, the regular stripes and the defect stripe are added together, and the spectrum is also the sum of the two spectra, as shown in Figure 2c. The proposed method filters only the center magnitude spectrum, the frequencies of the feature in a certain direction are selected to reconstruct the original image. The result shows that, by filtering the spectrum this way, the defect feature in the reconstructed image is substantially suppressed. Figure 2d–g shows more examples with different shapes of defects and directions. It is noticeable that, in Figure 2e, simulation of the horizontal repeating stripes, the defect feature in the reconstructed image, is more apparent. This is because the defect stretches over a large proportion in the horizontal direction; the spectrum of the feature has more magnitude than the smaller defects. In our scenario, however, the defects are always within one PV cell, and adjacent defects can hardly combine to become large enough to affect the performance; the features in different directions should thus be considered, as the features in the PV module are grids rather than stripes. The solution is to keep only one line in the spectrum. If horizontal stripes are required, then only the center column with a specific frequency is selected. Figure 2g shows the improvements in defect suppression using this technique.

### 2.3. PV Module Defect Detection

Defects can be easily extracted by subtracting the reconstructed image from the feature map after preprocessing. The process has two stages, as shown in Figure 1b. First, the vertical dividing lines are reconstructed by filtering the spectrum horizontally. Then the dividing lines can be used to segment the PV module into 12 cells. After segmentation, the spectrum is filtered within each PV cell vertically to reconstruct the horizontal busbars. The selection of the spectrum is based on prior knowledge of the number of PV cells and the number of busbars. Since there are 12 PV cells in a PV module, and 10 slices in one PV cell, the spectrum is filtered by maintaining the column index, which is a multiple of 12 in the center row, and the all-row index, which is multiple of 10 in the center column. 

The proposed method combines adaptive thresholding and the Fourier analysis method from the sections above, along with some other techniques, to achieve the goal of detecting cracks, breaks, and speckles. In this section, detailed implementation of the method is described with real EL image demonstrations.

#### 2.3.1. Preprocessing

**Figure 3:**
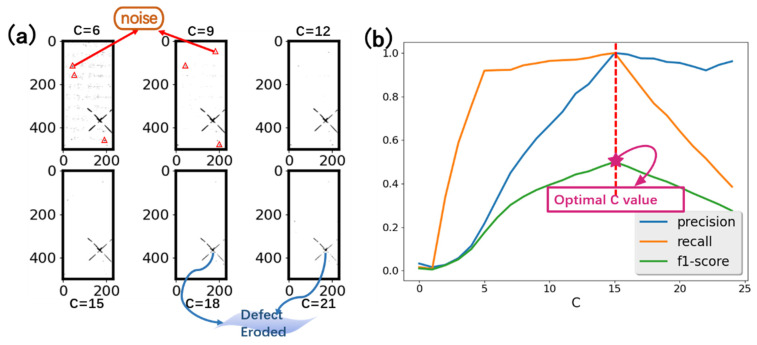
(**a**) detected defect pixels under different values of C. Some false detections are highlighted with red a circle. With a higher C value, true defects are eroded. (**b**) Precision, recall and f1-score change according to C value upon a training set of 20 samples.

Adaptive thresholding is used in the preprocessing step to extract all features, including the dividing edges, busbars in the cell, contours, and defects. Notice that, in our work, the grid electrode features are eliminated in preprocessing, as they are quite small relative to the scale of the entire PV module. A local threshold constant, C, is needed in this step to decide to which extent the features are kept. A higher C value will generate more features, but will harm the precision of defect detection. A lower C value will keep fewer features, but will promote precision and harm the recall rate. Figure 3a shows the defect pixels detected under different C values. In our design, the C value was chosen by calculating the f1-score as a trade-off between precision and recall. A training set with known defect pixel positions was used to select the C value. Different C values were used to preprocess the training set to generate the defect predictions. Therefore, the precision and recall rate of detection is calculated as:

$$\mathrm{Precision}=\frac{\mathrm{TP}}{\mathrm{TP}+\mathrm{FP}}\;$$

$$\mathrm{Recall}=\frac{\mathrm{TP}}{\mathrm{TP}+\mathrm{FN}}$$

where TP is the detected pixels that are indeed defects; TP + FP are all detected pixels; TP + FN are all ground truth defect pixels. Then, the f1-score is derived as:

$$F1=\frac{\mathrm{Precision}\times \mathrm{recall}}{\mathrm{precision}+\mathrm{recall}}$$

The training set contains 20 randomly selected EL images, with defective and normal ones, and the f1-score is calculated by setting different C values to detect defects. Figure 3b shows the trend of the f1-score with the C value. It can be seen from the graph that when C < 5, the proposed method cannot detect defects properly, so the precision and recall are both very low. When C > 5, the method can effectively detect defect; a higher C value will give better precision, until, at some point, there are not many false alerts though the ground truth defects begin to be obscured. It can be determined that, with C = 15, the f1-score is the best. Hence, this value was selected for the preprocessing.

#### 2.3.2. Reconstruction, Segmentation and Defect Locating

**Figure 4:**
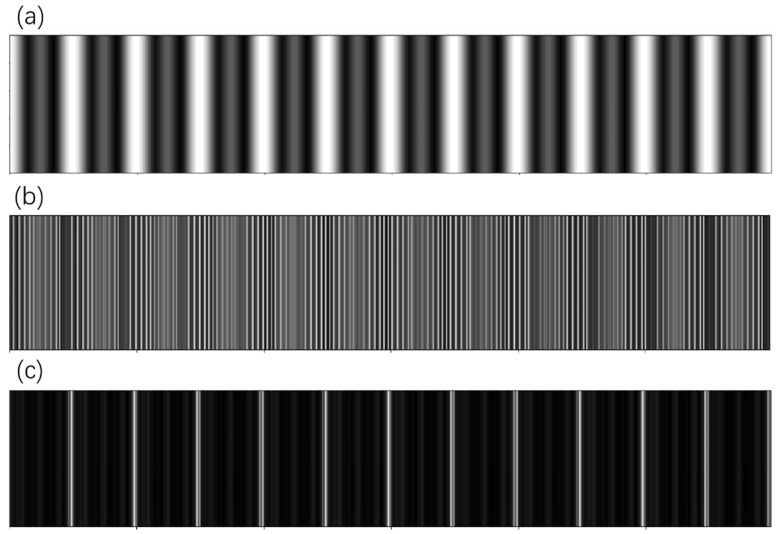
(**a**) reconstruction with only the first harmonic; (**b**) reconstruction with the wrong frequency selection; (**c**) reconstruction with all harmonics of frequency T = 12.

After preprocessing, the proposed method first rebuilds the periodic vertical dividing lines that repeat horizontally. The sought feature is the spectrum of frequency 12 along the horizontal direction. Figure 4 shows the spectrum of a real PV module image. The central point, which represents the mean value of the image, has the highest value in the spectrum. While, in the horizontal direction, there are a series of points with a high value. The first high-energy point has the coordinate of [0, 12] in the spectrum. The rest are multiples of 12, and have decaying energy. These frequencies are essential for reconstruction. It is recommended to reconstruct images with all the harmonics, otherwise, the reconstructed image will be more like a sinusoid signal, which is different from the desired feature, as shown in Figure 4. The frequency must be carefully selected to reconstruct the correct features, as the wrong frequency will cause problems for the result. Luckily the layout in our case was fixed, so the frequency was constant. One may experiment with the frequency if unsure of the value.

**Figure 5:**
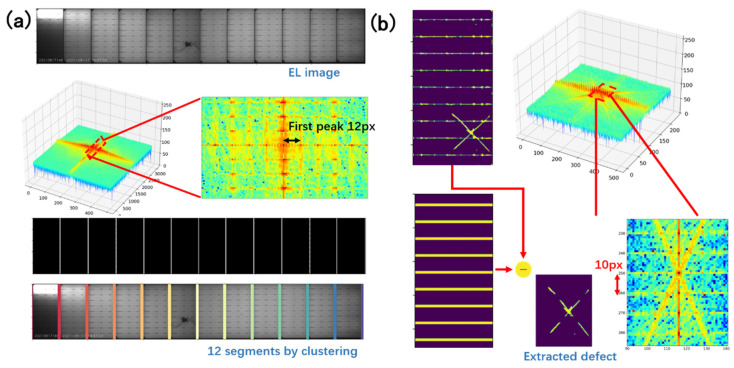
(**a**) The resulting spectrum of the Fourier transform, with the origin point shifted to center; (**b**) vertical reconstruction and defect extraction process.

The reconstructed image can be used as reference template, or the PV module can be segmented into cells for further processing. Usually, identify which cell has defects is required, and segmentation can be done on the reconstruction feature. However, segmentation is not trivial, though the groups are quite obvious in the reconstructed image. The aim is to group the white pixels into 12 (or 11) vertical lines, using the given center of clusters. Since the image may have a random shift in the horizontal direction, it is unclear whether 12 or 11 vertical lines have been reconstructed. DBSCAN is an unsupervised clustering algorithm that does not need the number of clusters; however, its time consumption is significant. In our implementation, we used K-means with a grid search to find the best match of clusters. The clustering results are shown in Figure 5a. 

The clusters that were aggregated from the reconstructed image could then be used to slice the PV module into 12 PV cells. In each cell, Fourier reconstruction was performed again while maintaining vertical frequencies of 10 and all harmonics. The reconstructed image will only contain the busbars, as shown in Figure 5b. The defect is all features that do not belong to the busbars.

## 3. Experiments and Evaluation

**Figure 6:**
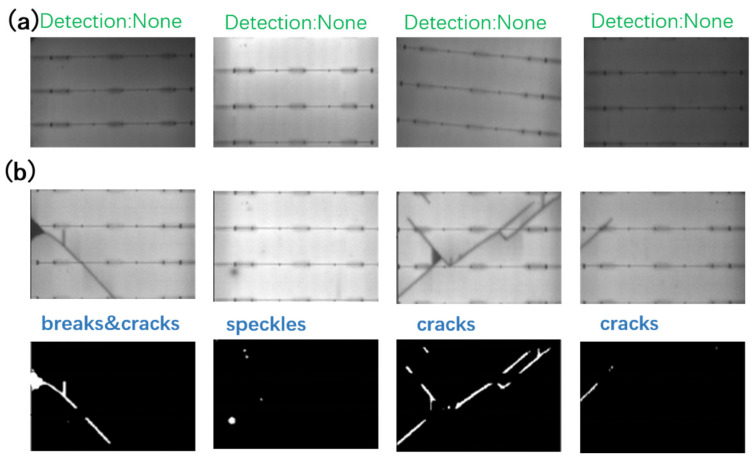
(**a**) Defect-free samples, the method is robust for illumination variations among different samples; (**b**) detected defects and located pixels.

The proposed method was tested on a set of PV-module EL images, including defect-free samples and defective samples, with all the above-mentioned types. The experiments were carried out on a laptop with an Intel(R) Core (TM) i7-8550U CPU @ 1.80 GHz 2.00 GHz processor. The EL images were around 400 × 3000 pixels with a variation of, at most, 20 pixels in width as a result of stochastic placing and cropping. The images had an 8-bit gray scale depth. The input of the system was the original-sized EL image. The system output the pixels of the cracks, breaks, and speckles. The average processing time for one 400 × 3000 EL image was about 0.13 ms, which was sufficiently fast for online detection. Figure 6a shows a set of defect-free PV cells with varying illumination conditions. No pixels are detected as defects in these images. Figure 6b shows a few samples with different shapes of cracks, breaks and speckles.

The entire experiment comprised 1000 EL images, 600 of which were defect-free samples, the rest included all three kinds of defects. The images were first manually examined and labeled. Defective samples included 254 samples with cracks, 174 samples with breaks, and 145 samples with speckles. Many samples had multiple defect types. The proposed method, with the adaptive thresholding parameter (C) set to 15, horizontal frequency set to 12, and vertical frequency set to 10, identified all the labeled defects with no misses. The method yielded 14 cases of false alarms, which were further affirmed as missed defects by humans. The proposed method outperforms human inspectors in terms of both accuracy and speed.

## 4. Discussion

The evaluation results show that the proposed method can find defect features regardless of layout interference. The brightness variation is solved by adaptive threshold preprocessing. This method relies on the periodic feature of layouts and the sparse feature of the defects. Since defects exist within one PV cell, a sparse characteristic is usually guaranteed from an entire PV module perspective. While it is possible that defects in multiple PV cells appear in same shape and location, which will break the sparse-defect hypothesis, this situation was never the case in our collected dataset. The method shows satisfying detection precision, and is suitable for PV-module defect detection in situations where no descent labeled training set is available. The method can also be applied in other applications, where a regular texture exists in the image. The detected defects in this method also formed a high-quality labeled dataset, which may serve as a training set for supervised learning studies. In future works, the results of the proposed method can be utilized as a PV defect dataset. 

## 5. Conclusions

In this paper, a defect detection method based on the Fourier reconstruction of periodic features is proposed. The proposed method is capable of detecting cracks, breaks, and speckles in the PV module EL image. The EL image can expose intrinsic defects that are not visible from the appearance. The periodic characteristic of the layout feature is exploited to distinguish defects from the normal edges. An adaptive thresholding step is adopted, which can robustly extract all features, despite the inhomogeneous illumination condition of the PV cells. The thresholding parameter is statistically generated with a few labeled samples, so no hand-engineered magic number is required. Based on the reconstructed features, as a reference template and segmentation result, defect pixels can be located.

The experimental results show that the proposed method can detect required defects in real-time, with good precision. The method is used in real PV-module testing pipelines.
